# Improving Digital Measurement-Based Care Within Emerging Adult Mental Health Care Pathways: Protocol for a Co-Design Phase Embedded in a Multimethod Rapid Learning Health System Study

**DOI:** 10.2196/93038

**Published:** 2026-09-11

**Authors:** Geneca Henry, Courtney Habina, Leanne Stamp, Melanie Fersovitch, Karen Moskovic, Lia Norman, Karina Pintson, Melissa Potestio, Julia Hews-Girard, Haley M LaMonica, Frank Iorfino, Paul D Arnold, Gina Dimitropoulos

**Affiliations:** 1 Department of Psychiatry Cumming School of Medicine University of Calgary Calgary, AB Canada; 2 Mathison Centre for Mental Health Research and Education University of Calgary Calgary, AB Canada; 3 Hotchkiss Brain Institute University of Calgary Calgary, AB Canada; 4 Recovery Alberta Calgary, AB Canada; 5 Faculty of Social Work University of Calgary Calgary, AB Canada; 6 Faculty of Nursing University of Calgary Calgary, AB Canada; 7 Brain and Mind Centre The University of Sydney Sydney, New South Wales Australia; 8 Alberta Children’s Hospital Research Institute University of Calgary Calgary, AB Canada

**Keywords:** co-design, dBMC, digital measurement-based care, emerging adults, mental health, participatory design, patient-reported outcome measures, PROMs, rapid learning health system, RLHS, transdiagnostic stratification, user-centered design

## Abstract

**Background:**

Emerging adults (EAs), typically ranging from 18 to 29 years, navigate a transitional period marked by rapid developmental, social, and psychological change. Despite heightened vulnerability to mental health (MH) concerns during this stage, service systems are often fragmented, with gaps between adolescent and adult care streams that leave many EAs without developmentally appropriate support. In response, developing approaches such as transdiagnostic stratification, which structures care around shared symptom processes and informs treatment intensity, and digital measurement-based care (dMBC), based on routine patient-reported outcome measures (PROMs), have gained traction but remain challenging to implement consistently. This reinforces the need for rapid learning health system (RLHS) approaches that leverage continuous data and feedback for ongoing improvement, as well as co-design (CD) methods that meaningfully integrate EA perspectives into service improvement.

**Objective:**

This research protocol describes a CD phase of an implementation study integrated into an RLHS, aimed at developing implementation resources to support the ongoing use of dMBC within EA MH services. Anticipated resources include clinician-facing workflow supports, guidance for using client-reported data in clinical decision-making, EA-oriented materials to support engagement with measures, and implementation planning resources to support uptake across the care pathway. Each co-designed resource will be developed to function across core stages of care, including intake, therapy, and treatment discharge.

**Methods:**

We used a concurrent multimethods design, integrating quantitative and qualitative approaches within a dual methodological framework combining user-centered design with participatory design to structure the CD process. EAs, clinicians, and clinical service leaders participated in a series of semistructured CD sessions to identify implementation needs and inform the development and refinement of implementation resources. Data collection and analysis began in January 2026 and are anticipated to conclude in November 2026.

**Results:**

As of August 24, 2026, 17 emerging adults and 20 clinical staff participants remained enrolled in the co-design phase. Both emerging adult cohorts had completed 6 co-design sessions, while clinical staff groups had completed 5 sessions, with final resource review ongoing. Data analysis and implementation resource finalization are expected to be completed by November 2026, with findings anticipated for publication in 2027.

**Conclusions:**

This study phase is expected to demonstrate the value of integrating CD within an RLHS to advance more responsive, contextually grounded dMBC implementation in EA MH care, while also contributing insights that can strengthen future CD efforts with this population.

**International Registered Report Identifier (IRRID):**

DERR1-10.2196/93038

## Introduction

### Background

Emerging adults (EAs) face elevated risks of mental health (MH) challenges [1], yet service systems often struggle to provide care that is both timely and suited to their developmental needs [2-4]. Emerging adulthood, defined as the transitional phase between adolescence and adulthood, typically spans 18 to 29 years [5], prominently characterized by 5 normative features: identity exploration, instability, self-focus, feeling-in-between, and possibilities/optimism [6,7]. This life stage coincides with the highest onset rates of mental illness (eg, mood and anxiety disorders and substance use disorders), with approximately 75% of lifetime disorders emerging before age 24 years [1]. The combination of ongoing neurobiological maturation, shifting social roles, independence, and increased academic and economic pressures contributes to this heightened vulnerability to MH disorders [7-10].

### Service Delivery Gaps for EAs

Despite clear evidence of need, MH systems remain poorly structured to serve this population, resulting in a substantial treatment gap [8,11]. The division between child/adolescent (hereafter, “youth”) and adult service systems often leaves EAs without proper care, as many age out of youth programs yet are not well supported by adult services that are not designed with their developmental needs in mind [8,11]. A recent systematic review [8] indicates that transition pathways in Canada are largely uncoordinated and that governmental supports are insufficient, contributing to inconsistent access to services at a time when many EAs require continuous care. The review also identifies a lack of national or provincial strategies that recognize emerging adulthood as a distinct period, creating uneven service delivery across regions and weak integration among care providers. These structural gaps limit the adoption of evidence-informed approaches (eg, community-based service delivery and electronic-MH initiatives) and leave many EAs without timely or developmentally appropriate care [8]. MH presentations in this age group tend to shift over short periods and often do not fit diagnostic categories created for younger or older populations [12], which complicates assessment and treatment planning, particularly when delays occur between initial contact and the start of care [13-15].

At the same time, EAs may seek a more active role in their MH care, and opportunities to take part in treatment decisions contribute to their transition toward greater autonomy in adulthood [14]. However, many services continue to prioritize clinician-led decision-making, which limits opportunities for individualized engagement [16,17] and often affects EAs more than other ages [18]. Assessment practices reinforce this pattern, such that systems that often rely on single-score distress measures, combined with clinical judgment, may not fully capture the complexity of EA needs or preferences [19]. These structural factors intersect with broader barriers related to stigma, financial concerns, poor MH literacy, and limited service availability, including long wait times [14,15]. Consequently, many EAs disengage from care altogether [14], with service use declining by as much as 50% to 70% during this critical transition period [20].

### Emerging Implementation Solutions and Their Barriers

Stratified care has emerged as a promising model for addressing systemic gaps through a more personalized and adaptive approach to MH service delivery [21]. Traditional MH service models are often shaped by program structures and organizational contexts, such that evidence-based interventions are delivered in relatively fixed ways that reflect service design and availability rather than being readily adapted to clients’ changing needs [22]. Stratified care, in combination with structured use of standardized assessment data, contributes to this shift by identifying an individual’s position along the care pathway and guiding changes in intervention intensity [23,24]. Integrating a transdiagnostic framework further strengthens this approach by focusing on shared symptom processes across conditions rather than discrete diagnoses [12,25-28]. Transdiagnostic interventions draw on strategies, such as targeting emotion regulation skills or reducing avoidance patterns, allowing them to be applied across co-occurring difficulties [26,29]. This process-focused approach helps stratified care models respond to overlapping needs as clients move through care. This combined approach is particularly relevant for EAs, as it accommodates developmental changes and social contexts that shape their help-seeking and engagement, thereby addressing gaps that often emerge as young people transition between youth and adult service systems [24]. Hereafter, stratified care will refer to this combined approach.

Digital measurement-based care (dMBC) is a relatively new, evidence-based approach [23,30] that can facilitate the development of streamlined, stratified, and transdiagnostic models in MH care [24,28]. This approach uses digital MH (dMH) platforms, such as the Australian web-based platform “InnoWell” [23], to systematically collect and apply patient-reported outcome measures (PROMs) that quantify self-reported symptoms, functional status, and well-being over time to guide treatment decisions [31-33]. PROMs are structured tools that capture clinically relevant information directly from clients and translate it into data to inform care in real time [34]. dMH platforms select PROMs that reflect key areas of MH and are grouped into domains such as everyday functioning, anxiety, psychological distress, and suicidal thoughts and behaviors. The core of dMBC involves the digital administration and automatic scoring of PROMs, which quantify symptom patterns that help identify and continuously monitor underlying transdiagnostic processes [23,31,32]. Clients and clinicians can review and track this data through interactive dashboards that show change over time [23,31,32]. Recent work shows that these platforms enhance youth and EA engagement when clinicians actively integrate the collected data into session discussions and treatment decision-making, allowing young people to better understand their symptom patterns and remain actively involved in their care [33,35].

Despite these advances, substantial implementation challenges continue to limit the reach and sustainability of dMBC within youth and EA MH services. System-level barriers commonly include variability in technology infrastructure, limited staffing resources, and the absence of clear organizational processes for integrating digital tools into routine workflows, which limit consistent use of dMBC across settings [36-39]. Digital platforms also introduce technical and procedural constraints when they do not integrate smoothly with existing electronic health records, creating additional or duplicative work for clinicians and reducing the feasibility of sustained adoption [36]. Clinician-level challenges include concerns about the time required to incorporate PROM data into sessions, uncertainty about interpreting digitally collected information, and apprehension that structured measures may constrain clinical dialogue or obscure the complexity of EA presentations [2,37,39]. For young people, barriers arise when measures feel repetitive or disconnected from their care experience, when the purpose of routine measurement is unclear, or when concerns about privacy and sensitive content lead to disengagement [2,40]. Items related to suicide risk introduce further complexity [28]. EAs may feel distressed when responding without real-time support, and clinicians often express concerns about liability and the operational pressures of responding to risk notifications [28,38,41,42]. These challenges illustrate that emerging solutions do not always translate automatically into effective implementation and that the success of dMBC depends on systems that can integrate dMH tools into everyday practices in ways that account for workflow realities, clinical judgment, and the lived experiences of young people accessing MH services. Future publications from our research will expand on these implementation barriers.

### Integrating Rapid Learning Health Systems and Co-Design for Responsive Implementation

dMBC implementation can be strengthened through a rapid learning health system (RLHS) framework [24], which leverages routinely collected health data to evaluate outcomes and guide ongoing improvements in service delivery [43,44]. This framework creates a continuous feedback cycle in which information from everyday clinical practice is analyzed and applied to enhance the quality and precision of care. Drawing on data from diverse real-world populations allows the RLHS to support adaptive learning and closer collaboration among clinicians, clients, and researchers [44-46]. A current study led by Dimitropoulos et al [24] is actively assessing the feasibility and acceptability of implementing an RLHS to support dMBC practices in stratifying and supporting EAs in their care pathways. This research is ongoing, and results have not yet been published. Nevertheless, embedding dMBC within an RLHS framework may not only be a practical integration of 2 evidence-based models but also a necessary step toward creating a more responsive and sustainable system of care.

Integrating co-design (CD) within an RLHS could further optimize dMBC implementation by positioning EAs and clinical service providers as active co-collaborators in identifying system barriers and developing service-level solutions. CD uses structured participatory techniques to elicit experiential knowledge and is regarded as an ethical and pragmatic approach because it positions those directly involved in a service context as active contributors whose insights inform the development or improvement of interventions that are responsive to real-world conditions [47,48]. Although dMH platforms, such as InnoWell, were initially informed by young people during development, there is limited research involving them together with clinicians in improving the actual delivery systems that support ongoing use of dMBC once the technology is introduced into clinical practice [35,49]. Consequently, decisions about how dMBC is introduced, maintained, or adapted often proceed without EA perspectives and can result in processes that may not fully reflect their lived experiences or the developmental factors that influence engagement [35]. Embedding CD in an RLHS addresses this gap by creating opportunities for EAs and MH service providers to participate in the continuous refinement of dMBC practices, promoting system-level changes informed by the realities of those who rely on these services and those who deliver them.

### Present Study and Objectives

This study protocol outlines the current phase of the ongoing RLHS implementation study by Dimitropoulos et al [24]. The phase was initiated in January 2026 and is expected to conclude in November 2026. A CD approach is currently applied to engage EAs, clinicians, and clinical service leaders in co-developing practical implementation resources to support the integration of dMBC into routine care. These resources are intended to improve the feasibility, acceptability, and usability of dMBC for both EAs receiving care and the clinical staff responsible for delivering it. To achieve these aims, we distinguish between primary and secondary objectives that address different aspects of the CD process (Textbox 1). The primary objectives focus on the intended outcomes of the CD work, whereas the secondary objectives examine the quality of the engagement process that supports those outcomes. This distinction enables this phase to assess not only what is produced through the CD but also how the participatory process affects collaboration, inclusivity, and the overall integrity of the findings. Our objectives reflect Canada’s Strategy for Patient-Oriented Research, which promotes active patient and stakeholder engagement to generate outcomes that are relevant, feasible, and applicable to improving care [50].

**Co-design phase objective types and descriptions.**

**Primary objectives**

- To identify a set of key domains that can be measured at distinct stages of the care pathway (eg, intake, assessment, therapy, and discharge) to generate clinical value for emerging adults (EAs), clinicians, and service delivery systems.
- To identify how EAs and clinicians would like the results of the validated measures to be shared and used at different points within the care pathways.
- To explore how EAs and clinicians prefer clinically complex or potentially emotionally sensitive measures to be integrated at various stages of the care pathway.
- To collaboratively develop a clear, actionable implementation plan for piloting co-design (CD) solutions for digital measurement-based care (dMBC), and to identify the resources, roles, and timelines associated with each step.
- To share outcomes and outputs from the CD process and elicit final feedback and reflections from the group. 

**Secondary objectives**

- To assess participants’ expectations, motivations, and readiness for involvement in the dMBC CD process, as well as the support they require to participate fully.
- To evaluate participants’ experiences during each CD session and explore how they perceived their ability to contribute, be heard, and influence the process.
- To examine the overall impact of the CD study on participants and ensure their insights directly inform the development of feasible and context-specific strategies for implementing dMBC in EA mental health services.
- To identify strengths and areas for improvement in the CD process to inform subsequent sessions within this study and shape future CD initiatives.

## Methods

### Study Context

Our study is one of several research projects conducted through the Framework for Research in Emerging Adults (FREA), a clinical research initiative led by the University of Calgary in partnership with Alberta Health Services that aims to transform the care and long-term health of EAs. Our study uses a concurrent mixed methods implementation design to evaluate dMBC integration into routine care while supporting continuous learning and iterative refinement of implementation practices. This study is ongoing and is anticipated to be completed in mid-2029.

To inform this CD phase, data collected between 2023 and 2025, including quantitative implementation data (eg, dMBC platform use, PROM completion rates, and longitudinal dMBC data collected between baseline and up to 12 months) and qualitative interviews and focus groups with EAs, clinicians, and clinical service leaders, were analyzed. Collectively, these findings identified consistent opportunities to strengthen the introduction, integration into clinical workflows, and use of dMBC during treatment to support care and clinical decision-making. Similar implementation needs were also identified through the study’s principal investigator’s (PI) parallel qualitative implementation research [2,36,37,41,42,51-53], which examined dMBC across various youth and EA MH care settings under different care models within Alberta between 2020 and 2025, providing the foundation for the current CD phase.

### Study Setting and Service Context

This FREA study is conducted at 2 outpatient MH clinics in Calgary, part of Alberta’s provincially coordinated health care system, including 1 specialized and 1 community-based clinic. Both clinics operate at a level 3 (high-intensity community-based services) according to the Level of Care Utilization System (LOCUS) developed by the American Association of Community Psychiatrists [54]. This means that these clinics provide services for individuals with moderate to high clinical needs who require intensive treatment but still can be appropriately managed in a community setting [54]. Both clinics offer comprehensive assessment, individual and group psychotherapy, psychiatric consultation, and coordinated referral pathways to facilitate continuity of care.

Prior to March 2026, dMBC under a stratified care model was implemented at both clinics using the InnoWell platform. The platform contained 23 biopsychosocial domains (eg, overall MH, depressed mood, and suicidal thoughts and behaviors), each linked to 1 or more corresponding PROMs used to assess that domain. Additional details on the domains [24] and the platform [23] are described elsewhere. Clinicians reviewed PROMs collected at 4 clinical time points: baseline, 6 and 12 months, discharge, or intervals selected by clinicians. EAs were also encouraged to use the platform at their own discretion. Following the conclusion of InnoWell use, both clinics have paused active dMBC implementation while the research team and clinic partners explore other dMBC platforms and use CD findings to adjust the implementation approach.

Care delivery at both clinics follows the same stratified care model; however, implementation may vary across sites. Although both clinics use the same overall pathway, differences may exist in how intake assessments are conducted, how information is incorporated into stratification decisions, and how clinical service leadership supports and oversees clinicians’ use of dMBC. At both clinics, EAs first participate in a fundamentals of change (FOC) psychoeducational group before undergoing treatment stratification. Information collected from the FOC group, intake assessments (including clinicians’ own assessment practices and, when available, dMBC PROM baseline scores), and clinician evaluations inform stratification decisions. Based on this, EAs are allocated to either a low-intensity (approximately 6-8 sessions) or high-intensity (approximately 12-14 sessions) psychotherapy stream, with access to additional services, such as psychiatry or group therapy, offered as needed. Once stratified, primary treatment clinicians use dMBC PROM scores collected at clinical time points, alongside clinical judgment, to monitor progress and adjust the intensity or direction of care as needed.

### Research Team and Reflexivity

The CD phase is led by a multidisciplinary research team with expertise in youth and EA MH, implementation science, and community-based research. The core team includes the study PI, 2 research coordinators, 1 postdoctoral researcher, and 3 research assistants. Three implementation leads from both this FREA study and the parallel dMBC implementation study have contributed to early-phase activities, including participant recruitment, early session material development, and co-facilitating several CD sessions. All team members have several years of experience in implementation research through sustained involvement in the study PI’s research program. With the exception of the postdoctoral researcher, the core team members were also involved in the parallel dMBC implementation study, providing familiarity with the implementation context, participating service settings, and experiences of clinicians and EAs throughout implementation. The team’s diverse educational and professional backgrounds contribute complementary perspectives throughout the CD process. Recognizing that these experiences, prior involvement in the parallel study, and interactions with participants may shape facilitation and interpretation, we engage in regular reflexive postsession debrief meetings and weekly team meetings. These discussions are used to consider how our perspectives shape facilitation and interpretation as the study phase evolves, thereby keeping the CD process grounded in participant contributions rather than researcher assumptions.

### CD Phase Design

**Figure 1:**
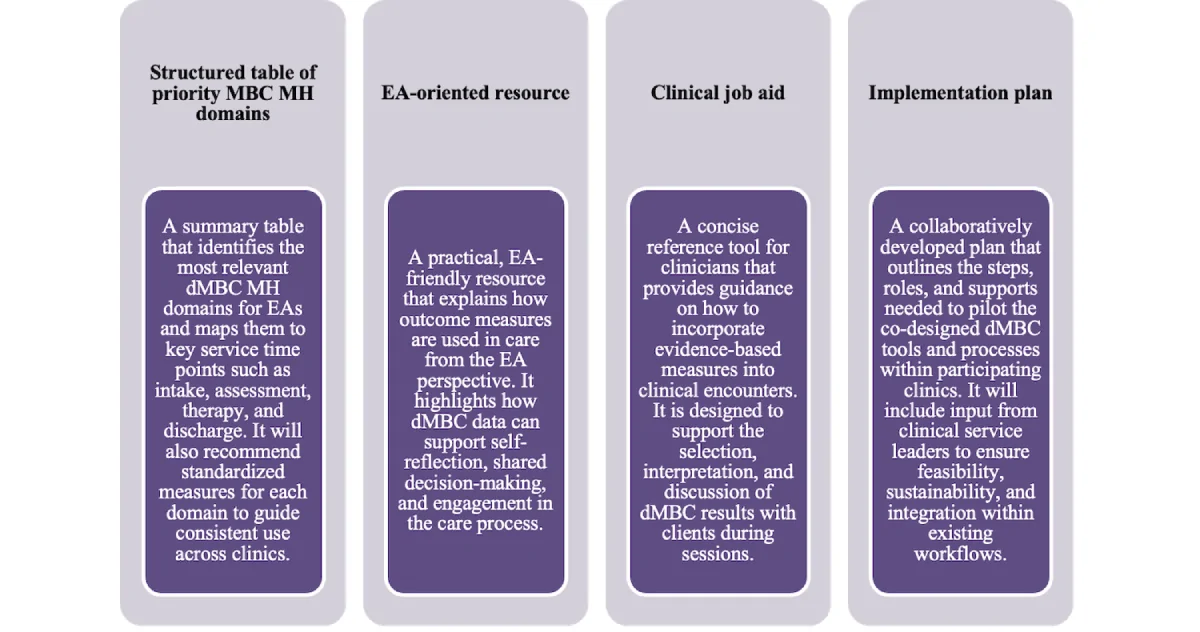
Four key co-designed implementation resources. dMBC: digital measurement-based care; EA: emerging adult; MBC: measurement-based care; MH: mental health.

The CD phase adopts a concurrent multimethod design that integrates qualitative CD activities with quantitative process evaluation data collected throughout the study. The CD process consists of a series of semistructured interactive sessions organized around the study’s 5 primary objectives and guided by the American Psychological Association’s measurement-based care (MBC) Professional Practice Guidelines [55], which outline the core competencies for administering measures, reviewing data collaboratively, and integrating MBC into ongoing treatment decisions. The sessions also address the guideline’s future directions by focusing on how MBC can be improved in practice, particularly using decision-support tools and attention to its application to EA MH care. Through this process, 4 key implementation resources will be developed to operationalize these priorities and provide concrete materials for piloting in the study’s next phase (Figure 1).

### Methodological Frameworks

We draw on 2 methodological frameworks that combine user-centered design (UCD) [56] and participatory design (PD) [57,58]. Integrating these frameworks establishes a structured yet adaptive foundation for our study, consistent with participatory approaches in implementation science. This integration facilitates a process that maintains methodological rigor while remaining relationally and contextually grounded. It supports the systematic development of the implementation resources as evidence-informed tools that reflect the practical and experiential realities of EAs and clinical staff involved in dMBC implementation.

### UCD

The foundation of UCD is grounded in established frameworks that emphasize a systematic understanding of end users’ needs, goals, and contexts through intentional, structured engagement [56]. End users are conceptualized as individuals who interact with or are directly affected by a system or process [56], consistent with how PROM users are commonly described [59,60]. This definition will serve as the basis for identifying end users in this study and establishes the foundation for their involvement in the CD process. According to Gulliksen et al [56], the effectiveness of UCD depends on users’ meaningful participation throughout all stages of design, ensuring that their experiential knowledge informs both conceptual development and practical implementation. The design process is inherently iterative, characterized by cycles of feedback, testing, and refinement that enable responsiveness to contextual and emergent challenges. Sustained engagement also requires adequate resources and a deliberate effort to integrate user experiences with organizational and operational considerations. Within this framework, end users are positioned as active collaborators whose insights shape how systems are conceptualized, developed, and evaluated.

The UCD principles are applied through our CD sessions, in which participants collaborate with the research team to generate and refine practical outputs to support the implementation of dMBC in real-world MH settings. This approach preserves UCD’s emphasis on iterative development and responsiveness to user needs.

### PD

We adopt the core principles of PD to guide our approach, focusing on its foundational commitment to equitable collaboration and shared decision-making among stakeholders [57,58]. While we are not implementing the full traditional PD methodology, which typically involves multiple cycles of piloting an intervention and reconvening groups for iterative refinement, we integrate its essential structured approaches. These include specific protocols for dialogue, consensus building, and iterative feedback that ensure all participants have clearly defined opportunities to contribute. These methods actively mitigate hierarchies that could silence less powerful voices. This framework positions EA clients, clinicians, and clinical service leaders as active collaborators rather than consultees, with each group contributing expertise rooted in their respective roles: clients with lived experience, clinicians in care delivery, and decision-makers in organizational implementation. PD principles specifically address inherent power differentials through structured opportunities for reciprocal dialogue and shared decision-making [58]. We use adapted techniques inspired by cooperative prototyping and facilitated dialogue sessions that enable participants to challenge assumptions and contribute equally to solution development [57].

This approach ensures that our co-designed implementation resources reflect multiple perspectives and remain grounded in both experiential and operational realities. The PD framework provides a structured process for validating ideas across participant groups, which helps balance client-centered priorities with the practical requirements of clinical feasibility and organizational sustainability [58]. When applied throughout the CD process, PD promotes the development of interventions that are meaningful to clients while remaining feasible to implement within existing health care systems [57].

### Participant Eligibility Criteria

Eligible EA participants must be aged between 17 and 26 years and either actively receiving or have been discharged within the previous 12 months (starting from when active EA recruitment begins) from one of the participating clinics. All eligible EAs will have previously used the InnoWell platform as part of their care at the clinic. Clinicians and clinical service leaders will participate together as a single clinical staff group at each site, as both roles contribute complementary clinical and operational perspectives on the implementation and use of dMBC. Conducting site-specific clinical staff groups ensures that discussions reflect each clinic’s local workflows, implementation practices, and organizational context. Clinical staff will be eligible if they are currently providing EA MH services at a participating clinic. Although prior involvement with the implementation of InnoWell before its conclusion is desirable, it is not required. New clinical staff who will be involved in ongoing dMBC implementation are also eligible to participate.

### Recruitment and Sample Size

The planned group composition and size were informed by focus group methodology [61], which recommends organizing participants according to shared characteristics, levels of expertise, or relative power related to the topic under investigation. Groups of approximately 5 to 10 participants are generally recommended to facilitate meaningful discussion while ensuring a diversity of perspectives. Accordingly, 2 types of CD participant groups are being implemented. EAs and clinical staff participate in separate groups to minimize potential power dynamics, particularly because some clinicians may be current or former providers of participating EAs.

The original protocol proposed 1 cross-site EA cohort of approximately 5 to 10 participants and 2 site-specific clinical groups of approximately 5 to 10 participants (10-20 clinical staff participants overall). Following commencement of the first EA cohort, the protocol was amended to recruit a second EA cohort to account for participant withdrawals and anticipated attrition across the multiple scheduled CD sessions over the summer months, increasing the planned EA sample from 1 cohort (5-10 participants) to 2 cohorts (10-20 participants). This sample size was further informed by the concept of information power [62], which suggests that qualitative studies with focused aims and participants possessing highly relevant experience may require fewer participants to generate information-rich data. Because participants were recruited based on their direct experience with dMBC at the participating clinics and contributed across multiple CD sessions, the planned sample size was considered appropriate to address the study phase’s objectives.

A purposive sampling strategy [63] was used to recruit participants who met the eligibility criteria. EAs were recruited from a pool of approximately 150 individuals who had previously consented to be contacted for future research participation. Invitations were distributed by telephone, email, and/or SMS text message. Clinical staff were introduced to the CD phase during scheduled team meetings at their respective sites in December 2025, followed by research team follow-up in January 2026 to provide additional study information and confirm participation. Recruitment of the first EA cohort occurred between February and March 2026, with 9 participants enrolled. Before the first CD session, 2 participants withdrew. Recruitment of the second cohort occurred between March and May 2026, resulting in 11 additional participants. To date, 1 participant has withdrawn from each cohort. Clinical staff recruitment resulted in 12 participants at one site and 8 at the second site. Initial recruitment exceeded the recommended focus group size to account for anticipated attrition throughout the longitudinal CD process. As the CD process remains ongoing, attendance varies across sessions due to participant availability, while session sizes remain consistent with recommended focus group sizes.

### CD Sessions

#### Overview

The clinical staff CD series commenced in early 2026, with one site beginning on January 27, 2026, and the other on February 5, 2026. Clinical staff had previously participated in study-related knowledge translation activities in November 2025 that introduced emerging findings on dMBC and facilitated discussion about service delivery considerations. The EA CD series commenced with a mandatory 1-hour orientation session, with cohort 1 beginning on April 29, 2026, and cohort 2 on May 20, 2026. The orientation introduced the purpose and process of CD, ensuring participants were fully informed about their roles, participation expectations, and the anticipated time commitment. The session also reviewed confidentiality procedures and included a brief interactive icebreaker activity to foster comfort and trust among participants. EA cohorts are scheduled to meet approximately every other week, whereas clinical staff sessions are scheduled less frequently to accommodate team availability and clinical responsibilities. The final EA sessions are expected to conclude by late August, with the clinical staff series by early October.

The series follows the 5 primary objectives in order, with each session focusing directly on 1 objective (Table 1). Sessions consist of semistructured discussions and interactive, group-based activities tailored for clinical staff and EAs to reflect their respective roles within the care pathway. The structure and duration of sessions for each objective continue to evolve iteratively throughout the CD process, allowing the research team to adapt the number and content of sessions based on participant engagement, emerging discussion, and group dynamics. Each participant group completes a minimum of 5 sessions, each lasting about 2 hours, delivered either virtually through a videoconferencing platform (Zoom) [64] or in person at the participating clinics. Depending on progress toward each session objective, 1 or more additional sessions may be conducted to provide sufficient time to complete the planned activities, further explore emerging topics, and collect adequate data to address the study’s primary objectives.

**Table 1:** Co-design series breakdown.

Session focus	Primary objective	Suggested activities
Prioritizing MH^a^ domains	To identify a set of key domains that can be measured at distinct stages of the care pathway (eg, intake, therapy, and discharge) to generate clinical value for EAs^b^, clinicians, and service delivery systems.	Breakout discussions on domains most and least meaningful to measure across the pathway Voting activity on domains most valuable at each stage
Using measures meaningfully	To identify how EAs and clinicians would like the results of the validated measures to be shared and used at different points within the care pathway.	Vignettes illustrating positive and negative client-clinician interactions with measures Breakout discussions on features, barriers, and enablers of effective versus poor measure integration
Using clinically complex measures meaningfully	To explore how EAs and clinicians prefer clinically complex or potentially emotionally sensitive measures to be integrated at various stages of the care pathway.	Vignettes illustrating effective and poor client-clinician interactions with emotionally challenging measures Breakout discussions on strategies, barriers, and facilitators for integrating sensitive measures into therapeutic encounters
Preparing to implement measures meaningfully	To collaboratively develop a clear, actionable implementation plan for piloting CD^c^ solutions for MBC^d^, and to identify the resources, roles, and timelines associated with each step.	Small-group mapping exercise to define key implementation outcomes, roles, timelines, and required resources Group sharing and feedback on feasibility, barriers, supports, and next steps Facilitated discussion to identify patterns, gaps, and collective commitments
Sharing the outcomes of the CD process	To share outcomes and outputs from the CD process and elicit final feedback and reflections from the group.	Co-led presentation of CD outcomes and outputs by participants and the study team Gallery walk showcasing activity artifacts and visual summaries, with participant feedback using sticky notes Live poll capturing reflections and future priorities Letters to future participants offering advice or encouragement

^a^MH: mental health.

^b^EA: emerging adult.

^c^CD: co-design.

^d^MBC: measurement-based care.

#### Participant Attendance and Retention

Because participation spans multiple CD sessions, some variation in attendance is anticipated. Participants are encouraged to attend every session, and virtual reminders are distributed before each scheduled session. For EA participants, a research team member follows up with individuals who miss a session without prior notice to confirm their continued participation or withdrawal from the CD phase. EA participants who remain enrolled are offered the opportunity to complete planned activities that do not require group discussion or collaborative decision-making (eg, polls or ranking exercises) either during a one-on-one virtual meeting with a research team member or immediately before the subsequent CD session. Responses from those activities are incorporated into the corresponding analysis of the activity artifact. To preserve rapport and psychological safety, EA participants remain within their originally assigned cohort throughout the study. Clinical staff will remain in their respective site-specific groups. Given the limited availability of clinical staff for research activities, participants who miss a session are not contacted to complete any research activity outside of the scheduled group session. Participants who withdraw from the CD phase contribute data collected up to the point of withdrawal; no additional data are collected thereafter.

#### Facilitation

Each session is facilitated by 2 members of the research team with experience in focus group facilitation and is supported by a research assistant who serves as a dedicated note-taker. Additional research team members may attend, depending on the anticipated group size, to assist with facilitation and participant support. Facilitators use structured facilitation techniques to encourage participation and elicit diverse perspectives, such as open-ended questioning, probing and follow-up questions, clarifying participants’ comments, checking for agreement or differing perspectives, and small- and whole-group discussions [61]. Divergent perspectives are explored through additional discussion and clarification rather than resolved through consensus, recognizing that differences in experiences and viewpoints may provide valuable insight to inform the development of the co-designed implementation resources. Throughout each session, facilitators summarize key discussion points to verify that participants’ perspectives have been accurately understood and provide opportunities for participants to clarify, expand on, or refine their contributions.

Every session includes a designated distress support person from the research team, whom EA participants can contact privately if they experience distress during a session or afterward. The support person follows a standardized protocol, including facilitating contact with the participant’s clinic or directing them to appropriate crisis or emergency MH services, as needed. Additionally, prior to each session, EAs are informed of the topics to be discussed, so they can make an informed decision about whether to participate in that session or prepare for potentially sensitive discussions.

### Data Collection

#### Quantitative

Quantitative data are collected electronically via REDCap [65,66] or on paper at multiple points throughout the CD process to provide both contextual and evaluative insights into participant engagement. These surveys were not mandatory to complete but were encouraged. At the start of the first CD session, a demographic survey and an InnoWell usage survey were administered to establish baseline context. The demographics survey captures key personal or professional characteristics relevant to each participant group, while the InnoWell usage survey gathers information about participants’ familiarity with and experience using the dMBC platform.

To address the study’s secondary objectives, participant engagement is assessed across 3 stages of the CD process (preseries, during the series, and postseries; Multimedia Appendix 1 provides details on instruments). At the preseries (first CD session) and postseries (last CD session) stages, participants complete module B of the Public and Patient Engagement Evaluation Tool (PPEET) [67]. Module B is designed to assess experiences with ongoing or long-term engagement initiatives and provides a structured way to evaluate participants’ perceptions of inclusiveness, influence, communication, and overall satisfaction with engagement processes over time. This consists of 22 questions, including Likert-scale items to rate agreement with various engagement-related statements, followed by open-text questions that allow participants to share additional comments or feedback.

For the purposes of this study phase, module B is adapted for administration at the 2 time points. For the preseries stage, the tool was adapted through team discussion to establish a baseline measure of participant engagement, with slight adjustments to item wording to reflect participants’ expectations and their initial understanding of their role in the CD process. The postseries version retains the same core content while incorporating minor contextual modifications to reflect participants’ experiences throughout the CD process. It is shortened to 20 items by removing the 2 baseline-specific questions on participants’ role and duration of involvement in the clinic, as these remain unchanged throughout the study phase.

Following each CD session, except the first and last, participants complete a brief session-specific feedback survey to capture immediate reflections on their experience. This survey includes 4 items from the Group Session Rating Scale (GSRS) [68]. The GSRS was selected because it is routinely used within the participating clinics’ group therapy programs, making it a familiar and low-burden measure for participants. The items capture participants’ perceptions of relational safety, topic relevance, approach fit, and overall group experience. An extra open-ended question, created by the research team, has been included to encourage participants to share comments or provide additional feedback about the session.

If clinical staff missed the first session, they were provided with the REDCap link to complete the demographic questionnaire, InnoWell usage survey, and the preseries PPEET at the start of their subsequent CD session. Given that all CD research activities for clinical staff are intended to be completed during the scheduled session, no follow-up reminders are provided after sessions. Accordingly, because clinical sessions may be separated by several weeks, missed GSRS surveys from participants who attended a session will not be completed retrospectively at a subsequent session. Similarly, no follow-up reminders will be sent for missed postseries PPEET surveys.

Because the orientation session was mandatory, all EA participants received the demographic, InnoWell usage, and preseries PPEET surveys and were encouraged to complete them during the session. As EA participants have greater flexibility to conduct research activities outside scheduled sessions, a research team member contacted participants who did not complete these surveys by phone or email up to 3 times within 1 week to facilitate completion. The same follow-up procedure applies to EA participants who attend a CD session but do not complete the GSRS or postseries PPEET surveys.

#### Qualitative

The CD sessions form the primary qualitative dataset. Sessions are audio-recorded and transcribed verbatim to document participant dialogue and group interactions. Recordings are obtained through the Zoom platform for virtual sessions or an encrypted digital recorder for in-person sessions. Transcription is completed by a university-approved transcriptionist or the Zoom transcription feature, with participants reminded to avoid using identifying details. All transcriptions are verified by a member of the research team.

Each CD session also yields “activity artifacts,” which are physical or digital materials developed collaboratively through group activities or discussions [69]. They capture how participants collectively developed and organized ideas in relation to the CD phase’s primary objectives. Table 2 provides examples of potential activity artifacts that may emerge through these facilitator-guided activities.

All participants will be invited to take part in follow-up individual interviews or focus groups at the end of the CD series. These discussions will allow participants to reflect on their experiences and engagement with the CD process. The same data collection procedure used for the CD sessions will apply to these interviews. Data from these interviews will contribute to the secondary objectives of this study phase.

**Table 2:** Examples of co-design activities and resulting artifacts.

Activity	Prompts: clinical staff	Prompts: EAs^a^	Artifact (digital or physical)
Ranking: individual or collectively ordering of domains, ideas, or priorities according to importance or relevance	“Rank the domains that are the most clinically useful for decision-making.” “Order potential implementation strategies from most to least feasible within your workflow.”	“Rank the following domains based on how much they influence your current, past, or future treatment goals.” “From most to least helpful, order the ways you would like your clinician to share your measurement results.”	Screenshot of a shared digital workspace showing the final ranked list, combining individual or group rankings into a single prioritized sequence
Journey mapping: participants in breakout groups chart the care experience, identifying touchpoints for dMBC^b^ use	“Map where PROMs^c^ currently fit in your workflow. Where do they add or disrupt value?” “Where could dMBC strengthen continuity between sessions?”	“Draw your care journey, from first appointment to the most recent. Where do you think completing measures would be the most helpful? “When would feedback feel most motivating during care?”	Large paper or digital maps illustrating each breakout group’s care stages, barriers, and preferred PROM touchpoints
Vignette group discussion: group discussion using fictional clinician-client scenarios to explore issues and solutions	“How could the clinician in this vignette better introduce dMBC to support engagement?” “What could be changed in this scenario to improve workflow?”	“In this story, what could the clinician do differently to make feedback conversations more collaborative?” “What would make this process feel more supportive rather than evaluative?”	Annotated slides or shared notes summarizing strengths, challenges, and improvements for each vignette

^a^EA: emerging adult.

^b^dMBC: digital measurement-based care.

^c^PROM: patient-reported outcome measure.

### Data Analysis

#### Quantitative

Demographic data will be analyzed using descriptive statistics [70] to summarize participant characteristics (eg, age, gender, role in the study, and years of experience or involvement with the clinic). Frequencies and percentages will be reported for categorical variables, and measures of central tendency (eg, means and medians) and dispersion (eg, ranges and SDs) will be reported for continuous variables, as appropriate. The InnoWell usage survey will follow the same analysis method. Both results will be aggregated to describe the composition of the participant group, to provide context for interpreting the findings, and to examine the diversity of perspectives represented in the CD phase.

Preseries and postseries survey responses will be analyzed after the series concludes, using descriptive statistics. Likert-scale items will be treated as continuous data for descriptive purposes and summarized using means or medians, measures of dispersion, and frequencies to provide transparency in response distributions across the 4 domains (inclusiveness, influence, communication, and overall satisfaction). Open-text responses will be analyzed using qualitative content analysis to identify themes, elaborations, or concerns expressed by participants. Both survey datasets will be aggregated to compare and assess changes in participants’ perceptions of engagement throughout the CD process. Comparisons will focus on differences in Likert-scale scores and thematic differences in open-text responses, with subgroup analyses conducted where relevant (eg, EAs vs clinical staff).

Session-specific feedback surveys are analyzed after each CD session to inform planning for subsequent sessions and ensure participant feedback is addressed throughout the CD process. Each item of the GSRS measure is scored on a 0 to 10 scale, summed to produce a total score (0-40), and analyzed using descriptive statistics (means, medians, SDs, and ranges). Item-level and total scores are examined to assess participants’ perceptions of session quality across domains of relationship, goals/topics, approach, and overall experience. The open-text questions are analyzed using qualitative content analysis to identify recurring themes, explanations for ratings, and suggestions for improvement. Following completion of the CD series, GSRS scores and qualitative feedback will be aggregated to examine patterns in participant engagement and experiences throughout the CD process.

#### Qualitative

The CD session and interview recordings are analyzed using rapid qualitative analysis (RQA) [71,72] and a qualitative descriptive approach [73], which are suitable for implementation research and RLHS contexts, where findings must inform ongoing processes in a timely manner. RQA facilitates the systematic condensation of large, discussion-based datasets into structured summaries that preserve participants’ language and contextual meanings, thereby minimizing interpretative bias during analysis [71,72]. Several members of the research team are involved in this analysis, with each trained by the study PI or a senior research team member to use this RQA method. These members include the research coordinators and assistants. Textbox 2 provides an overview of the qualitative analysis workflow. All qualitative data analysis activities are expected to be completed by November 2026.

**Qualitative analysis workflow by analysis stage, timing, and analytic process.**

**Stage 1: develop structured session rapid qualitative analysis (RQA) summary (after each co-design [CD] session)**

- One research team member reviews the session transcript and prepares a structured summary documenting session discussion using participants’ language wherever possible and including relevant direct quotations. The summaries are organized by topics that naturally arose during the session rather than by a predetermined framework. A second reviewer independently compares the summary with the transcript, verifies its accuracy and completeness, and revises it as needed. Reviewer notes are documented throughout the process to capture any researcher interpretations or preliminary themes. The reviewers meet to discuss any differences identified during the verification process and resolve them through consensus. Where consensus cannot be reached, discrepancies are brought to the broader research team for discussion until consensus is reached.

**Stage 2: organize session findings** (**after each verified summary)**

- Verified summaries are entered into a summary matrix organized by participant group (emerging adult [EA] cohorts and clinical staff groups) and session-focused objective. Session artifacts (eg, worksheets, rankings, polls, and sticky notes) are retained as a separate qualitative data source and organized according to the corresponding session objective.

**Stage 3: review findings and determine next steps (after a participant group completes the planned sessions for a CD objective)**

- The primary and secondary reviewers do a brief review of the matrix summaries within the participant group and discuss preliminary findings in a team meeting. Alongside co-facilitators’ session observations, the research team discusses whether sufficient data have been generated to address the CD objective, recognizing that objectives may overlap across sessions. If activity incompletion and emerging topics require further exploration, additional sessions are warranted. Where only limited clarification is needed, these topics are integrated into the subsequent CD session objective rather than addressed through an additional session.

**Stage 4: conduct objective-level thematic analysis (after each participant group completes a session objective)**

- Summary matrices are synthesized using a qualitative descriptive approach [73] via thematic analysis [74]. One research team member identifies preliminary low-inference themes describing participant perspectives related to the session objective, and a second reviews and refines the thematic interpretation until consensus is reached. Agreed themes are further synthesized into an objective-specific RQA summary report for each participant group. A third research team member reviews the report before final review by the study principal investigator (PI). Any feedback provided by the third person and study PI is addressed by the team members involved in the thematic analysis. Any disagreements follow the same process as in the first analytic stage. Session artifacts are analyzed separately using the same qualitative descriptive thematic approach.

**Stage 5: develop overall thematic synthesis (following completion of all participant group CD sessions)**

- Themes developed for each session objective are integrated within each participant group to produce an overall thematic summary (including both objective-specific RQA and corresponding activity artifact reports). Participant group summaries are then compared to identify shared priorities and differing perspectives to finalize the co-designed implementation resources.

**Stage 6: end-of-CD-series interview RQA summary and thematic analysis (following completion of each participant group’s CD sessions)**

- Individual interview and focus group data will be analyzed using the same analytic workflow described in stages 1 to 5. Briefly, transcripts will undergo structured RQA, including independent verification and consensus, followed by integration into summary matrices and qualitative descriptive thematic analysis to identify key themes related to the study phase’s secondary objectives.

### Implementation Resource Development

Qualitative findings will be used to develop the implementation resources throughout the CD process. Resource content will be informed by shared understandings, recommendations, and priorities identified across participants, alongside divergent perspectives that provide meaningful insights for implementation. Throughout the CD process, the research team will collectively review and discuss emerging findings to determine which insights are appropriate and feasible to include in the implementation resources. Where perspectives differ between EAs and clinical staff, these distinctions will be retained and, where appropriate, clearly identified within the resources to ensure participant-group-specific recommendations are accurately represented.

As the implementation resources evolve, emerging content and unresolved questions will be brought back to the participants during subsequent CD sessions to gather feedback and guide further refinement. Additional implementation resources may also be developed if identified through the CD process.

The final CD objective may be completed over more than 1 session. During the initial session, draft versions of the resources may be presented to obtain participants’ initial feedback. We may then incorporate this feedback into revised drafts, which may then be emailed to participants as watermarked copies for internal review only. Participants may subsequently be invited to another review session to provide additional feedback on the revised resources. Implementation resources are expected to be finalized by November 2026.

### Quality Assurance

We hold weekly team meetings throughout the CD process to review phase progress, discuss implementation considerations from session findings, and monitor methodological consistency. Adaptations to session materials, facilitation approaches, and development of the implementation resources are discussed and documented during these meetings. Standardized facilitation materials and documented team decisions will support consistency and provide a transparent audit trail of iterative refinements.

### Dissemination Plan

Key findings generated through the CD process will be shared through future peer-reviewed publications, academic conferences, and targeted knowledge translation presentations to clinical partners. The finalized co-designed implementation resources will not be publicly disseminated following this CD phase. Instead, they will be piloted and evaluated within the participating clinics during the next phase of the implementation study before any broader dissemination is considered.

### Ethical Considerations

Ethical approval for the implementation study was obtained from the University of Calgary’s Conjoint Health Research Ethics Board (REB21-0616). The same ethics board also approved the CD phase (REB25-1537).

All eligible participants provided informed consent prior to joining the CD phase. Consent was either obtained electronically through REDCap prior to the first session or electronically or physically at the start of the first session. Only those who provided consent can participate in the CD phase. EAs aged 17 years were required to demonstrate full decision-making capacity prior to confirming eligibility. This was assessed by a member of the research team using a 4-item decision-making capacity questionnaire developed for the implementation study (Multimedia Appendix 2). To demonstrate adequate capacity to provide informed consent, minors must correctly answer all 4 questions.

Only EA participants will receive compensation for their time and contributions. Compensation is set at US $18.05 per hour of participation and will be distributed after each session, allowing EA participants to withdraw at any time without penalty and still receive compensation for sessions already attended. Clinical staff will not be compensated, as their participation is considered part of their professional responsibilities and organizational quality improvement efforts within their respective clinics. Participants can leave the CD phase at any time.

If requested, their quantitative data will be removed within 7 days of their withdrawal. However, this does not apply to data collected during group-based research activities (eg, CD sessions or focus group interviews). Given the interactive nature of these activities, individual contributions become integrated with those of other participants and may inform collaboratively generated materials, making it infeasible to isolate and remove an individual participant’s data after collection. This limitation has been communicated to all participants during the informed consent process and documented in the consent form.

All research data will be stored on secure University of Calgary servers using approved platforms, with transcripts deidentified so that participants are referenced only by study ID and role. Data access will be restricted to authorized research team members. Given the group-based nature of the CD sessions, absolute confidentiality cannot be guaranteed. To promote privacy and respectful participation, a confidentiality pledge will be reviewed at the outset, and participants will be reminded to use first names only and to share information they are comfortable disclosing. They will also be informed of the limits to confidentiality, including mandatory reporting requirements for potential risks of harm to self or others.

## Results

As of August 24, 2026, the CD phase remains ongoing, with the current status and implementation resources developed summarized in Table 3. Data analysis and implementation resource finalization are expected to be completed by November 2026, with findings anticipated for publication in 2027.

**Table 3:** Current status of the CD^a^ phase as of August 24, 2026.

Participant group and participants	Current CD status	Implementation resources developed
EAs^b^: a total of 17 participants enrolled across 2 cohorts (cohort 1: n=7; cohort 2: n=10). Attendance has varied across sessions.	Both cohorts have completed 6 CD sessions (objective 3 had been split into 2 sessions) and are in the final stage of the CD process. Three EA-focused implementation resources have undergone group review and initial refinement. Interested participants are currently independently reviewing the revised resources and providing additional feedback through individual review sessions with a research team member.	MBC^c^ guidebook for EAs: practical information to support EAs in understanding and using MBC throughout care. Frequently asked questions: concise responses to common questions about MBC, including measure completion, use of results, and privacy. Shared measurement plan: a collaborative tool to guide discussions between EAs and clinicians about how measurement will be used and individualized throughout care.
Clinical staff: a total of 20 participants across sites (site 1: n=12; site 2: n=8). Attendance has varied considerably across sessions due to staff availability.	Both site-specific groups have completed 5 CD sessions addressing the first 4 CD objectives (objective 4 had been split into 2 sessions). The final CD session, in which participants will review and provide feedback on 5 implementation resources, is currently being scheduled.	Recommended implementation guidelines for leadership: recommendations to support MBC integration within clinical workflows and service delivery. Priority list of domains with key considerations: ranked list of domains for measurement across the care pathway and considerations for their use. Practical MBC guide for clinicians: a practice-oriented job aid for integrating MBC into routine clinical care at the different stages of the care pathway. Measure scoring and interpretation reference guide: a standardized template that clinics can use to develop reference guides for measures selected for future use, with sections covering measure description, items and response options, scoring, clinical interpretation, and references.

^a^CD: co-design.

^b^EA: emerging adult.

^c^MBC: measurement-based care.

## Discussion

### Anticipated CD Phase Outcomes

A growing body of implementation research highlights that system-level decisions in EA MH often overlook the relational, experiential, and developmental factors that shape engagement during this life stage, contributing to the inconsistent uptake of dMBC despite evidence that routine PROM feedback strengthens clinical decision-making and outcomes [75,76]. This protocol responds to that gap by examining CD as a methodological approach that can strengthen the responsiveness of dMBC within an RLHS structure. CD is used to refine existing implementation processes in ways that center EA perspectives and address barriers that are commonly missed when decisions are made at organizational or clinical levels without sustained EA involvement. Embedding CD within an RLHS positions EAs as active contributors to iterative refinements, supporting the development of implementation strategies that evolve through continuous learning and reflect the complexities of EA service use. We anticipate that insights generated through this CD process will inform practical adaptations to dMBC implementation and demonstrate the value of CD in producing implementation resources grounded in participant contributions and suited to real-world care contexts. What is learned through this process will also guide refinements to our CD methodology in future work, particularly efforts to deepen EA engagement in research activities and amplify their influence in service improvement initiatives.

### Potential Strengths and Limitations

A key potential strength of this CD phase is our design, which supports iterative learning and responsiveness throughout the CD process, allowing implementation strategies and resources to evolve in ways that reflect the realities of EA MH care. The multimodal methodological framework guiding the CD sessions provides a structure that attends to the experiential and emotional dimensions throughout the implementation design, creating conditions for participants to contribute insights grounded in their lived experiences. The use of RQA and postsession feedback may further strengthen this approach, as these methods provide systematic ways to synthesize emerging insights and incorporate them into subsequent sessions, thereby fostering continuity and refinement throughout the CD process.

This work will also advance equity in MH implementation research by attending to populations whose developmental transitions and intersecting identities shape distinct experiences of access and engagement. Although RLHS models emphasize learning cycles that incorporate evidence, practice, and interest-holder perspectives, key perspectives remain underrepresented in service design [77]. Positioning equity as a guiding principle enables decision-making and adaptation that reflect the complexities of EA experiences and the varied clinical perspectives and expertise of participating staff. This approach treats equity as integral to our implementation study’s ongoing learning process rather than as an outcome assessed at the end of the study.

A potential limitation of this CD phase is the narrow range of variability in service contexts, which may limit the transferability of the findings. The organizational structures, priorities, and client needs represented in the participating clinics may not reflect those of other MH settings, particularly services operating in rural or remote regions or under different clinical or administrative models. Although this work is situated within an RLHS intended to support the translation of CD insights into implementation and technology-related refinements, some findings may remain shaped by local conditions rather than generalizable patterns across the broader system.

Another limitation is the use of separate participant groups for EAs and clinical staff. Although this approach was intentionally adopted to promote open discussion and minimize potential power imbalances, it may preclude direct dialogue between the groups and may limit opportunities to collectively explore and reconcile differing perspectives. To mitigate this limitation, we will present emerging findings from previous sessions and participant groups throughout the CD process to encourage consideration of alternative perspectives and inform the development of implementation resources that reflect the priorities of all participant groups.

Additionally, the use of the GSRS measure serves as another limitation. The measure was originally developed and psychometrically validated to measure therapeutic alliance in group therapy settings, specifically the client-leader-group relationship within clinical care, rather than participatory research or CD. However, given that our implementation study is situated within a clinical care setting and participants were already familiar with the measure through routine group therapy programs, the GSRS was considered an appropriate and pragmatic tool for capturing brief session feedback to support iterative refinement of the CD process. As the measure has not been validated for use in participatory research or CD settings, the findings should be interpreted as process feedback rather than a formal evaluation of participant engagement or the quality of the CD process.

### Future Directions

The next phase of this work will involve piloting the co-designed implementation resources. While the CD participants will not be reconvened during the pilot, the perspectives of EAs and clinical staff will continue to iteratively inform the process through future evaluative surveys, interviews, and focus groups. This will allow us to evaluate how the outputs function in practice and how they can be further refined.

Moreover, our future research will expand on the secondary objectives of this phase, which examine participants’ expectations, experiences, and the impact of involvement in the CD process. Insights from these findings will inform future CD initiatives on how to better support equitable participation and foster conditions that promote increased engagement and shared decision-making. This direction will guide the development of a new CD study involving EAs accessing MH services in postsecondary settings, where institutional and developmental contexts may influence participation differently. This will involve investigating how these factors can support a more evidence-informed perspective on adapting CD methods to promote meaningful collaboration and create conditions for participant perspectives to continue shaping the evolution of dMBC and other system-level innovations.

## Appendix

Multimedia Appendix 1 Quantitative instruments administered during the co-design phase.

Multimedia Appendix 2 Instrument for evaluating decisional capacity for minors.

## Abbreviations

CD: co-design
dMBC: digital measurement-based care
dMH: digital MH
EA: emerging adult
FOC: fundamentals of change
FREA: Framework for Research in Emerging Adults
GSRS: Group Session Rating Scale
LOCUS: Level of Care Utilization System
MBC: measurement-based care
MH: mental health
PD: participatory design
PI: principal investigator
PPEET: Public and Patient Engagement Evaluation Tool
PROM: patient-reported outcome measure
RLHS: rapid learning health system
RQA: rapid qualitative analysis
UCD: user-centered design

## Data Availability

The datasets generated or analyzed during this study are not publicly available to protect the confidentiality of research participants and the privacy of their clinical settings but are available from the corresponding author on reasonable request.
