# Supplementary material for: Improving Digital Measurement-Based Care Within Emerging Adult Mental Health Care Pathways: Protocol for a Co-Design Phase Embedded in a Multimethod Rapid Learning Health System Study
Source: JMIR Res Protoc. 2026 Sep 11;15:e93038. doi: 10.2196/93038 (PMC13615379; doi:10.2196/93038)
Supplement: Supplementary file 1 [file resprot_v15i1e93038_app2.pdf]

FREA Measurement-Based Care Co-design Study

INSTRUMENT FOR EVALUATING DECISIONAL CAPACITY FOR MINORS

Question	Score	Participant Response
**What is the purpose of this project?**<br><br>Correct Response: The participant expresses an understanding that the purpose of the project is to engage young people in a co-design process to determine which mental health domains are most important to measure in mental health care for emerging adults, and to co-design supportive resources and strategies to support the use of measures.	0= Incorrect<br><br>1= Correct	
**Do you have to be in this project if you don't want to?**<br><br>Correct Response: The participant expresses an understanding that their involvement in this study is their choice (completely voluntary) and they don't need to be in the study if they don't want to be.	0= Incorrect<br><br>1= Correct	
**If you decide to withdraw from this study, will it negatively impact any current or future mental health care that you receive?**<br><br>Correct Response: The participant expresses an understanding that withdrawing from this co-design study will not negatively impact current or future	0= Incorrect<br><br>1= Correct	

mental health care received, and that they can end their involvement in the study at any time with no repercussions.		
**What are three things that will happen if you participate in this research project co-design?**<br><br>Correct Response: The participant expresses an understanding that they will be asked to:<br>• Complete a survey to provide their demographic information<br>• Complete a series of short surveys about their expectations and experiences of participating in the co-design process<br>• Participate in a series of co-design sessions to share their perspectives and provide input about using measures in mental health services.	0= Incorrect<br><br>1= Correct	

Total Score: ___________

Note: The participant must have a perfect score of 4 to be eligible to participate. If they score less than 4, the information will be explained to the participant, and they will be asked again. If they do not score 4 after 3 attempts, they cannot participate.

If passed: *"Okay, great! You understand the co-design study and your role in it well and can give consent. I will send the demographic survey and consent form to you after the call. Please fill it out and send it back to me when you can. Do you have any questions?"*

If failed: *"Unfortunately, it doesn't seem that you are able to participate in this co-design study. Thank you for your interest and have a great rest of your day."*
