# Supplementary material for: Improving Digital Measurement-Based Care Within Emerging Adult Mental Health Care Pathways: Protocol for a Co-Design Phase Embedded in a Multimethod Rapid Learning Health System Study
Source: JMIR Res Protoc. 2026 Sep 11;15:e93038. doi: 10.2196/93038 (PMC13615379; doi:10.2196/93038)
Supplement: Supplementary file 2 [file resprot_v15i1e93038_app1.docx]

Co-Design Evaluation Tools

Framework for Research in Emerging Adults: Measurement-Based Care Co-Design Study

# **Contents:**

## Pre-Series Survey

## Post-Session Survey

## Post-Series Survey

# Pre-Series Survey

*[Administer at the end of the first session’s orientation presentation]*

*[All questions in this survey are from the Public and Patient Engagement Evaluation Tool (PPEET; Abelson & PPEET Research-Practice Collaborative, 2018)]*

**Pre-Series Survey**

**Name:** ________________________	**Date:** ________________________

We would like to hear about your expectations and perspectives before the subsequent FREA MBC Co-Design sessions begin. This survey has 22 questions. **You may skip any questions you do not wish to answer.**

Your responses are confidential and will be securely stored, accessible only to the study team. Please note that responses are **not** anonymous—they may be linked to your name or participant ID for analysis and follow-up, but this information will be handled carefully and in line with ethical guidelines to protect your privacy.

Your feedback is important to improving our engagement process. If you have any questions about this survey, please contact the study team at **emergingadults@ucalgary.ca**. Thank you for your participation.

**Background Information**

1. **What perspective do you bring to the MBC Co-design Study?**

- Emerging Adult
- Clinician
- Decision-Maker

2. **How long have you been with the Recovery Alberta clinic as an employee or client?**

- Less than 6 months
- 6-12 months
- 1-2 years
- 3-4 years
- 5 years or more

**Part A: Communication and Supports for Participation**

***Please indicate your level of agreement with each of the statements below.***

3. **I have a clear understanding of the purpose of the MBC Co-design Study.**

- Strongly disagree
- Disagree
- Neither agree nor disagree
- Agree
- Strongly agree

4. **The supports I need to participate in the MBC Co-Design Study are available.**

- Strongly disagree
- Disagree
- Neither agree nor disagree
- Agree
- Strongly agree

5. **I have enough information to contribute to be able to carry out my role.**

- Strongly disagree
- Disagree
- Neither agree nor disagree
- Agree
- Strongly agree

6. **What else would you like us to know about how participation in the MBC Co-Design Study will be supported?** *(Please write your answer below)*

**Part B. Sharing Your Views and Perspectives**

***Please indicate your level of agreement with each of the statements below.***

7. **I will be able to express my views freely.**

- Strongly disagree
- Disagree
- Neither agree nor disagree
- Agree
- Strongly agree

8. **I feel that my views will be heard.**

- Strongly disagree
- Disagree
- Neither agree nor disagree
- Agree
- Strongly agree

9. **A wide range of views on the topics that will be discussed will be shared.**

- Strongly disagree
- Disagree
- Neither agree nor disagree
- Agree
- Strongly agree

10. **The individuals participating in the MBC Co-Design Study represents a broad range of perspectives on the topic.**

- Strongly disagree
- Disagree
- Neither agree nor disagree
- Agree
- Strongly agree

11. **What else would you like us to know about how you will be able to share your views?** *(Please write your answer below)*

**Part C. Impacts and Influence of the Engagement Initiative**

***Please indicate your level of agreement with each of the statements below.***

12. **I think that the MBC Co-Design Study will achieve its objectives.**

- Strongly disagree
- Disagree
- Neither agree nor disagree
- Agree
- Strongly agree

13. **I am confident that the study team will take the feedback provided by the MBC Co-Design Study into consideration.**

- Strongly disagree
- Disagree
- Neither agree nor disagree
- Agree
- Strongly agree

14. **I think that the work of the MBC Co-Design Study will make a difference to the work of the clinic.**

- Strongly disagree
- Disagree
- Neither agree nor disagree
- Agree
- Strongly agree

15. **In your role, what influence do you think you will have?** (Please write your answer below)

16. **What else would you like us to know about the influence you think the MBC Co-Design Study will have?** (Please write your answer below)

**Part D. Final Thoughts**

***Please indicate your level of agreement with each of the statements below.***

17. **As a result of my participation in the MBC Co-Design Study, I will be better informed about measurement-based care.**

- Strongly disagree
- Disagree
- Neither agree nor disagree
- Agree
- Strongly agree

18. **Overall, I will be satisfied with this engagement initiative.**

- Strongly disagree
- Disagree
- Neither agree nor disagree
- Agree
- Strongly agree

19. **This engagement initiative will be a good use of my time.**

- Strongly disagree
- Disagree
- Neither agree nor disagree
- Agree
- Strongly agree

20. **What will be the strengths of the MBC Co-Design Study?**  *(Please write your answer below)*

21. **What could be improved so far about the MBC Co-Design Study?** *(Please write your answer below)*

22. **What else would you like us to know about your upcoming experience with the MBC Co-Design Study?** *(Please write your answer below)*

**End of Survey**

Thank you for your participation.

If you would like to connect with a member of the research team to discuss your experience with this activity further, please contact the study team at [**emergingadults@ucalgary.ca**](mailto:emergingadults@ucalgary.ca)

The Public and Patient Engagement Evaluation Tool has been licensed under a Creative Commons Attribution-NonCommercial-Share Alike 4.0 International License. ©2018, Julia Abelson and the PPEET Research-Practice Collaborative. McMaster University. All rights reserved.

# Post-Session Survey

*[Administer at the end of each co-design session]*

[*Part 1 is four questions from the Group Session Rating Scale (GSRS; Duncan & Miller, 2007) & Part 2 is an open-text question developed by the study team for comments or additional feedback]*

**Post-Session Survey**

**Name:** ________________________ **Date:** ________________________ **Session #:** _____________________

We would like your feedback on today’s FREA MBC Co-design session. This survey has five questions and asks about your experiences and suggestions. **You may skip any questions you do not wish to answer.**

Your responses are confidential and will be securely stored, accessible only to the study team. Please note that responses are **not** anonymous—they may be linked to your name or participant ID for analysis and follow-up, but this information will be handled carefully and in line with ethical guidelines to protect your privacy.

Your feedback is important to improving our engagement process. If you have any questions about this survey, please contact the study team at **emergingadults@ucalgary.ca**. Thank you for your participation.

**Part 1. Group Session Rating Scale (GSRS)**

***Please rate today’s group by placing a mark on the line nearest to the description that best fits your experience***

| I did not feel understood, respected, and/or accepted by the leader and/or the group. | **Relationship**  **I----------------------------------------------------------------------I** | I felt understood, respected, and accepted by the leader and the group. |
| --- | --- | --- |
| We did *not* work on or talk about what I wanted to work on and talk about. | **Goals and Topics**  **I----------------------------------------------------------------------I** | We worked on and talked about what I wanted to work on and talk about. |
| The leader and/or the group’s approach is a not a good fit for me. | **Approach or Method**  **I----------------------------------------------------------------------I** | The leader and group’s approach is a good fit for me. |
| There was something missing in group today—I did not feel like a part of the group. | **Overall**  **I----------------------------------------------------------------------I** | Overall, today’s group was right for me—I felt like a part of the group |
| International Center for Clinical Excellence  _______________________________________  [www.scottdmiller.com](http://www.scottdmiller.com)  © 2007, Barry L. Duncan and Scott D. Miller    Licensed for personal use only | | |

**Part 2. Comments and Additional Feedback**

**Do you have any additional comments, feedback, or suggestions you would like to share?** *(Please write your answer below)*

**End of Survey**

Thank you for your participation.

If you would like to connect with a member of the research team to discuss your experience with this activity further, please contact the study team at [**emergingadults@ucalgary.ca**](mailto:emergingadults@ucalgary.ca)

# Post-Series Survey

*[Administer at the end of the last co-design session]*

*[All questions in this survey are from the Public and Patient Engagement Evaluation Tool (PPEET; Abelson & PPEET Research-Practice Collaborative, 2018)]*

**Post-Series Survey**

**Name:** ________________________	**Date:** ________________________

We would like your feedback on your experience in the FREA MBC Co-design Study. This survey has 20 questions. Some ask you to rate your agreement with statements, while others invite comments or suggestions. **You may skip any questions you do not wish to answer.**

Your responses are confidential and will be securely stored, accessible only to the study team. Please note that responses are **not** anonymous—they may be linked to your name or participant ID for analysis and follow-up, but this information will be handled carefully and in line with ethical guidelines to protect your privacy.

Your feedback is important to improving our engagement process. If you have any questions about this survey, please contact the study team at **emergingadults@ucalgary.ca**. Thank you for your participation.

**Part A: Communication and Supports for Participation**

***Please indicate your level of agreement with each of the statements below.***

1. **I had a clear understanding of the purpose of the MBC Co-Design Study.**

- Strongly disagree
- Disagree
- Neither agree nor disagree
- Agree
- Strongly agree

2. **The supports I needed to participate in the MBC Co-Design Study were available.**

- Strongly disagree
- Disagree
- Neither agree nor disagree
- Agree
- Strongly agree

3. **I had enough information to be able to carry out my role.**

- Strongly disagree
- Disagree
- Neither agree nor disagree
- Agree
- Strongly agree

4. **What else would you like us to know about how participation in the MBC Co-Design Study was supported?** *(Please write your answer below)*

**Part B. Sharing Your Views and Perspectives**

***Please indicate your level of agreement with each of the statements below.***

5. **I was able to express my views freely.**

- Strongly disagree
- Disagree
- Neither agree nor disagree
- Agree
- Strongly agree

6. **I feel that my views were heard.**

- Strongly disagree
- Disagree
- Neither agree nor disagree
- Agree
- Strongly agree

7. **A wide range of views on the topics discussed were shared.**

- Strongly disagree
- Disagree
- Neither agree nor disagree
- Agree
- Strongly agree

8. **The individuals participating in the MBC Co-Design Study represented a broad range of perspectives.**

- Strongly disagree
- Disagree
- Neither agree nor disagree
- Agree
- Strongly agree

9. **What else would you like us to know about how you were able to share your views?** *(Please write your answer below)*

**Part C. Impacts and Influence of the Engagement Initiative**

***Please indicate your level of agreement with each of the statements below.***

10. **I think that the MBC Co-Design Study achieved its objectives.**

- Strongly disagree
- Disagree
- Neither agree nor disagree
- Agree
- Strongly agree

11. **I am confident that the study team takes the feedback provided by the MBC Co-Design Study into consideration.**

- Strongly disagree
- Disagree
- Neither agree nor disagree
- Agree
- Strongly agree

12. **I think the work of the MBC Co-Design Study makes a difference to the work of the clinic.**

- Strongly disagree
- Disagree
- Neither agree nor disagree
- Agree
- Strongly agree

13. **In your role, what influence do you think you have had to date?** *(Please write your answer below)*

14. **What else would you like us to know about the influence you think the MBC Co-Design Study will have?** *(Please write your answer below)*

**Part D. Final Thoughts**

***Please indicate your level of agreement with each of the statements below.***

15. **As a result of my participation in the MBC Co-Design Study, I am better informed about measurement-based care.**

- Strongly disagree
- Disagree
- Neither agree nor disagree
- Agree
- Strongly agree

16. **Overall, I was satisfied with this engagement initiative.**

- Strongly disagree
- Disagree
- Neither agree nor disagree
- Agree
- Strongly agree

17. **This engagement initiative was a good use of my time.**

- Strongly disagree
- Disagree
- Neither agree nor disagree
- Agree
- Strongly agree

18. **What are the strengths of the MBC Co-Design Study?**  *(Please write your answer below)*

19. **What could be improved about the MBC Co-Design Study?** *(Please write your answer below)*

20. **What else would you like us to know about your experience with the MBC Co-Design Study?** *(Please write your answer below)*

**End of Survey**

Thank you for your participation.

If you would like to connect with a member of the research team to discuss your experience with this activity further, please contact the study team at [**emergingadults@ucalgary.ca**](mailto:emergingadults@ucalgary.ca)

The Public and Patient Engagement Evaluation Tool has been licensed under a Creative Commons Attribution-NonCommercial-Share Alike 4.0 International License. ©2018, Julia Abelson and the PPEET Research-Practice Collaborative. McMaster University. All rights reserved.

# References

Abelson, J., & PPEET Research-Practice Collaborative. (2018). *Public and Patient Engagement Evaluation Tool (PPEET), version 2.0*. McMaster University. <https://ppe.mcmaster.ca/wp-content/uploads/2023/04/PPEET_Version-2.0_Full-Set.pdf>

Duncan, B. L., & Miller, S. D. (2007). *Group Session Rating Scale (GSRS)*. International Center for Clinical Excellence. <http://www.scottdmiller.com>
